# Epidemiological and molecular profile of breast cancer: a retrospective study in Casablanca, Morocco

**DOI:** 10.11604/pamj.2025.50.105.43868

**Published:** 2025-04-22

**Authors:** Khadija Khadiri, Zineb Khadrouf, Abderrahmane Mellouki, Oumaima Bouchra, Amina Essalihi, Abdallah Naya, Majda Taoudi Benchekroun, Mehdi Karkouri

**Affiliations:** 1 Laboratory of Immunology and Biodiversity, Faculty of Sciences Ain Chock, Hassan II University, Casablanca, Morocco,; 2 Department of Pathology, University Hospital Center Ibn Rochd, Casablanca, Morocco,; 3 Laboratory of Cellular and Molecular Pathology, Faculty of Medicine and Pharmacy, Hassan II University, Casablanca, Morocco

**Keywords:** Breast cancer, epidemiology, molecular profile, invasive carcinoma, Morocco

## Abstract

**Introduction:**

breast cancer is a major public health concern in Morocco due to delayed diagnosis and treatment, largely due to its high incidence. It is the most prevalent cancer in women. This study aimed to report the epidemiological and the molecular profile of invasive breast carcinoma using surrogate immunohistochemical markers.

**Methods:**

during 24 months from 1^st^ January 2020 to 31^st^ December 2021 at the Pathology Department of Ibn Rochd University Hospital of Casablanca, Morocco, we conducted a retrospective study comprised 1,558 breast cancer cases, including male patients. The generated data was analyzed using GraphPad Prism 8 software to define the epidemiological and molecular features of breast cancer.

**Results:**

this epidemiological study reveals that the mean age of patients was 52 ± 12.27 years. The most common histological type is invasive breast carcinoma of no special type, which accounts for 90.5% (n=1,410) of cases. Regarding Ellis and Elston modified Scarff-Bloom-Richardson (SBR) grade, grade III was the most common, 52.2% (n=521), followed by grade II, 44% (n=438), and grade I, 3.8% (n=38), as assessed by the SBR grade. The molecular classification results indicate that luminal B was the most common subtype at 52.2% (n=441), followed by triple-negative at 19.6% (n=166), with HER2 phenotype representing 17.4% (n=147) and luminal A at 10.8% (n=91).

**Conclusion:**

this study highlights that invasive breast carcinoma of no special type is the most common type, with grade III tumors being the most frequent. The majority of cases were luminal B, underscoring the need for targeted therapeutic strategies.

## Introduction

Breast cancer is a major public health concern worldwide, accounting for 11.7% of all cancers, all sites combined, and for 24.5% of female cancers in 2020 [1], it is the fifth cause of cancer-related deaths with an estimated number of 2.3 million new cases worldwide according to the GLOBOCAN 2020 data [2]. In Africa, the breast cancer mortality rate of 19.4% is the highest worldwide, rendering the disease a critical public health issue in the majority of African countries [3]. The incidence of this malignant tumor is increasing in all regions of the world, but the highest incidence occurs in industrialized countries. Almost half of the cases on a global scale are in developed countries compared to Asian and African countries [2,4,5]. In Morocco, breast cancer is the most frequently diagnosed malignancy and the leading cause of cancer-related mortality among women. In 2022, it accounted for 38.8% of all cancer cases, with 12,756 new cases reported exclusively in females. Additionally, breast cancer represented 20.1% of all cancers diagnosed across genders and age groups [6].

Breast cancer diagnosis and treatment remain a significant challenge in oncology due to the disease´s heterogeneous nature. Breast tumors exhibit substantial variability in clinical presentation and therapeutic response, necessitating the development of multiple classification systems, clinical, histopathological, and molecular, to facilitate precise identification of aggressive subtypes and guide therapeutic decision making. Among these, molecular classification has proven particularly valuable, as it enables oncologists to tailor treatment strategies based on tumor biology [7]. The assessment of tumor biomarkers plays a crucial role in differentiating molecular subtypes of breast cancer, thereby aiding in prognosis prediction and therapeutic response assessment. Key biomarkers, including Estrogen Receptors (ER), Progesterone Receptors (PR), Human Epidermal Growth Factor Receptor 2 (HER2), and proliferation markers such as Ki67, are essential in guiding treatment selection. Hormone receptor (HR)-positive tumors benefit from hormone therapy, while HER2-positive cases require anti-HER2 targeted treatments. By classifying breast cancer at the molecular level, clinicians can achieve a more accurate prognosis evaluation and implement personalized therapeutic strategies, ultimately improving patient outcomes [8,9]. Understanding the epidemiological patterns and molecular characteristics of breast cancer is essential for optimizing therapeutic approaches [10].

This study aimed to analyze the epidemiological and molecular profile of breast cancer using surrogate immunohistochemical markers in patients followed at the Ibn Rochd University Hospital of Casablanca, Morocco.

## Methods

**Study design and setting:** this is a 24-month retrospective study conducted from 1^st^ January 2020 to 31^st^ December 2021, aimed to investigate the demographic, and immunohistochemical characteristics of invasive breast cancer in Moroccan patients. Data was extracted from the files of the Pathology Department of Ibn Rochd University Hospital of Casablanca. Ibn Rochd University Hospital of Casablanca is the largest health facility in Morocco and, the Pathology Department serves as a major diagnostic hub for cancer cases, including breast cancer, with access to well documented patient records and histopathological reports, the setting provides a rich source of retrospective data for analyzing breast cancer characteristics, treatment patterns and outcomes.

**Study population:** the study comprises 1,558 cases of invasive breast carcinoma, including male patients. Inclusion criteria specified all histologically confirmed cases of invasive breast carcinoma, excepting lesions, tumors of benign origin (adenofibromas, cysts, hyperplasias...), non-epithelial malignancies, and histologically unconfirmed cases.

**Laboratory analysis:** to assess the tumor biomarkers expression (ER, PR, Ki67, and HER2), we employed immunohistochemistry (IHC), a laboratory technique used to detect specific antigens in tissue samples using antibodies, it combines immunological and histochemical methods to visualize proteins of interest, aiding in disease diagnosis, particularly in cancer. We used the ER-EP1 clone, PR-PgR636 clone, Ki67-MIB1 clone on Dako Autostainer Link 48, and the HER2-4B5 clone on the Ventana BenchMark GX automated system.

**Data collection:** the data is collected electronically using the Laboratory Information System (LIS). Reports are identified through the use of a unique patient number for all patients who have been diagnosed and confirmed with invasive breast cancer, and entered into Microsoft Excel (2019). Data on age, sex, histological type, expression of molecular biomarkers, and molecular subtypes were collected.

**Definitions:** in the present study, key variables were methodically defined for the purpose of accurate data collection. Patient age and sex were recorded, while the histological type was classified in invasive carcinoma of the no special type (NST), infiltrating lobular carcinoma (ILC), and invasive micropapillary carcinoma. Tumor biomarker expression was assessed using immunohistochemistry, with estrogen receptor and progesterone receptor considered positive if at least 1% of tumor cells showed staining. HER2 status was classified according to standard guidelines (0+, 1+, 2+, 3+), and Ki67 was measured as a percentage in order to assess tumor proliferation. The molecular subtypes of breast cancer were then categorized on the basis of these markers: Luminal A, Luminal B, HER2-enriched, and triple-negative.

**Statistical analysis:** data were analyzed using GraphPad Prism 8, a statistical software integrating scientific graphing, statistical analysis, and data organization into a single platform, making it particularly valuable for biomedical research. The variables were expressed as percentages of the total number of cases. A descriptive analysis of proportions was conducted to evaluate epidemiological and molecular patterns. The distribution of breast cancer cases was analyzed based on demographic, histological, and immunophenotypic characteristics. The findings were subsequently compared with national and international reports to identify potential variations and trends in the epidemiological and molecular profiles of breast cancer.

**Ethical considerations:** the study protocol was conducted under the ethical principles, the ethical committee of Ibn Rochd University Hospital of Casablanca approved this study on April 28, 2023, under reference 13/2022.

## Results

**Epidemiological characteristics:** the study incorporated 1,558 individuals who had been detected with breast cancer. On average, these patients were 52 ± 12.27 years old. The age group most commonly impacted by breast cancer ranged from 40-49 to 50-59, which amounted to 31.8-31% (n=458, n=448) of all instances of cancer, between 60-69 with a percentage of 17.5% (n=252), cases ≥70 represent 7.1% (n=102). By contrast, the incidence of breast cancer among individuals aged under 30 years was lower at 1% (n=15). Almost all of the affected patients, approximately 98.8% (n=1539), were female, while males represented only 1.2% (n=19).

**Histological characteristics:** in terms of histology, invasive carcinoma of the no special type is the most common, accounting for 90.5% of cases (n=1410), while infiltrating lobular carcinoma (ILC) at just 3.7% (n=58), invasive micropapillary carcinoma (IMPC) is rare, representing only 1.7% (n=26) of cases. Regarding SBR grade, the proportion of grades SBR II and III is relatively high, estimated at 44% (n=438) for SBR II and 52.2% for SBR III (n=521). Conversely, only 3.8% (n=38) of cancer cases were classified as grade I (Table 1).

**Table 1:** distribution of cases by histological characteristics of breast carcinoma

Histological characteristics	Frequency	Percentage (%)
Histological type (n=1558)		
Invasive carcinoma of no special type	1410	90.5%
Infiltrating lobular carcinoma	58	3.7%
Invasive micropapillary carcinoma	26	1.7%
Other	64	4.1%
Histological type grade SBR* (n=997)		
SBR I	38	3.8%
SBR II	438	44%
SBR III	521	52.2%

* SBR: Ellis and Elston modified Scarff-Bloom-Richardson

**Figure 1:**
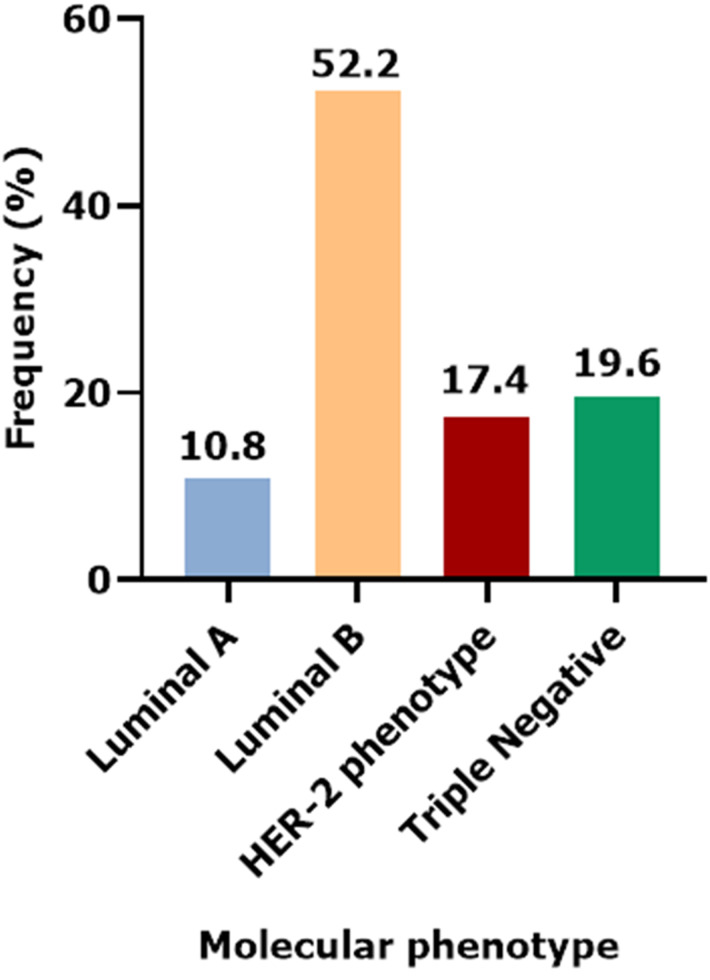
distribution of cases by molecular phenotype

**Immunophenotypic characteristics:** the immunohistochemical profile displayed that 66.1% (n=876) of tumors were ER+PR+, 25.2% (n=334) ER-PR-, 8.2% (n=109) ER+PR-, and 0.5% (n=7) with ER-PR+. Analysis for HER2 overexpression indicated that score 0+ represents 32.8% (n=407), score 1+ 27.4% (n=341), score 2+ 19.1% (n=237), and score 3+ 20.7% (n=257) (Table 2). The evaluation of the Ki67, a tumor biomarker used to estimate cell index, showed that 81.5% (n=1070) of the cases exhibited a value greater than or equal to 20% (proliferation index is high). Immunohistochemistry testing was performed to determine the ER, PR, and HER2 status of the tumors. This test indicated four biological subtypes. In our data, 845 cases were classified according to their molecular phenotype. The study illustrates that 10.8% (n=91) of tumors belong to luminal group A, 52.2% (n=441) to luminal group B, 17.4% (n=147) to HER2, and 19.6% (n=166) exhibit a triple-negative pattern (Figure 1).

**Table 2:** distribution of cases by immunophenotypic characteristics of breast carcinoma

Immunophenotypic characteristics	Frequency	Percentage (%)
Estrogen and progesterone receptors status (n=1326)		
ER+PR+	876	66.1%
ER-PR-	334	25.2%
ER+PR-	109	8.2%
ER-PR+	7	0.5%
Human epidermal growth factor 2 score (n=1242)		
0+	407	32.8%
1+	341	27.4%
2+	237	19.1%
3+	257	20.7%

ER: estrogen receptors; PR: progesterone receptors

## Discussion

Our study aimed to analyze the epidemiological and molecular characteristics of breast cancer in Moroccan patients, focusing on the histological type, expression of tumor biomarkers, and the molecular subtypes. Our results revealed that invasive carcinoma of no special type was the most common histological type (90.5%). The most tumors were high-grade SBR III 52.2%, with a high proliferation index Ki67 ≥20% in 81.5% of cases, hormone receptor analysis showed that 66.1% were ER+PR+, HER2 overexpression was observed in 20.7% of cases. Molecular subtyping identified luminal B as the most prevalent subtype (52.2%).

Our research findings are consistent with the literature, particularly the work of Khanfir *et al*. in 2022 [11], who highlight that invasive carcinoma of no special type is the primary and most prevalent histological form, surpassing all other types and accounting for more than half of all invasive carcinomas. However, invasive lobular carcinoma (ILC) constituted only 3.7% of the cases in our study, which is consistent with the literature [12]. Regarding the SBR grade, our study revealed the predominance of SBR grade III, which suggests that our study population has a higher proportion of more aggressive forms of breast cancer, these findings are inconsistent with Fouhi *et al*. Moroccan study in 2020 [13], which revealed the predominance of grade II, with a percentage of 55.9%, followed by grade III 32.6%. The results of our study were inconsistent with those reported in the literature, which could be explained by the larger sample size in our study compared to that of Fouhi *et al*. study, this difference in sample size may have influenced the distribution of variables. The predominance of high-grade tumors in our study suggests that a large portion of the breast cancer cases in the Moroccan population may have aggressive, poorly differentiated tumors.

Hormone estrogen receptors are indicators of tumor differentiation, whilst PR positivity signifies ER functionality [13]. In our research, the hormone receptor tested positive in 66.1% of cases. The study of these prognostic factors is crucial, as the receptor´s expression is a good prognostic element and, most importantly, predictive of the response to hormonal treatment [14]. These findings align with the existing Moroccan study, with Abbass *et al*. in 2011 [15]. It is worth noting that breast carcinogenesis is not solely tied to the hormonal pathway, as other activation pathways also play a role.

This present study also observed a predominance of HER2-negative tumors, suggesting that a significant portion of breast cancer cases in our population lack overexpression of the HER2 protein. These results are in accordance with Ngo *et al*. finding in 2017 [16], that 73.3% of cancer cases are HER2 negative. Ki67, another tumor biomarker used to estimate cell proliferation in breast cancer, was found to be elevated in 81.5% of cancer cases during our study. Based on these findings, it can be concluded that the majority of breast cancer cases exhibit high levels of Ki67, indicating rapid tumor growth and acting as a negative prognostic factor for breast cancer [17].

Immunohistochemical analysis of the ER, PR, and HER2 status in tumors allowed the identification of four molecular subtypes (luminal A, luminal B, triple-negative, and HER2). Each subtype has unique molecular characteristics. Our study revealed a higher prevalence of luminal B compared to luminal A, HER2, and triple-negative subtypes. These findings align with Serrano-Gomez *et al*. study in 2016 [18], and with Fouhi *et al*. study in 2020 [13]. They demonstrated that luminal B group has the highest frequency, with the distinction observed in the other groups. Identifying the molecular characteristics of breast cancer can lead to a better prognosis by predicting the probability of survival and recurrence in patients. This information allows for to assessment of the risk of disease progression and adjust treatment accordingly.

The limitations of this study include limited access to clinical patient data, which may limit the scope of the analysis and the representativeness of the findings. Additionally, the lack of follow-up regarding therapeutic responses restricts the ability to assess the long-term efficacy of treatments and the correlation between molecular profiles and patient outcomes, such as recurrence and survival. The strengths of this study lie in the sample size of our study, its detailed focus, and the valuable data on the epidemiological aspects of breast cancer, combined with a thorough analysis of histological subtypes and immunophenotypic markers (ER, PR, HER2 and Ki-67). This approach enables accurate predictions of prognosis and the tailoring of treatment strategies based on tumor characteristics, leading to more personalized and effective breast cancer management.

## Conclusion

This present study described the epidemiological and molecular profile of breast cancer among the Moroccan patient population. Our findings were in line with the literature, showing that no special type carcinoma is the most prevalent histological type, while luminal B is the most represented molecular phenotype. A thorough understanding of histopathological classification and epidemiological characteristics is essential for individualizing treatment strategies, optimizing therapeutic decision making, and improving prognostic accuracy. These findings provide valuable insights for refining breast cancer management, supporting the development of targeted therapies and evidence-based prevention strategies to enhance patient outcomes in Morocco.

### What is known about this topic

- Breast cancer is the most prevalent cancer in women, both globally and in Morocco;
- In Morocco, breast cancer is the most frequently diagnosed malignancy and the leading cause of cancer-related mortality among women;
- The luminal subtype is the most prevalent molecular phenotype of breast cancer worldwide.

### What this study adds

- Our findings advance the understanding of breast cancer patterns in Morocco, providing valuable data for prognosis and guiding personalized treatment approaches.
- Our study showed, luminal B is the most prevalent invasive breast cancer phenotype in Morocco, suggesting a potentially more aggressive disease progression in our population.
- A higher prevalence of breast cancer in the 40-49 and 50-59 age groups of Moroccan women compared to older women ≥60.
